# High-sensitivity C-reactive protein and cyclothymic temperament as predictors of suicidal risk

**DOI:** 10.1192/j.eurpsy.2025.2025

**Published:** 2025-08-26

**Authors:** L. Cavallo, G. Longo, L. Orsolini, U. Volpe

**Affiliations:** 1 Unit of Clinical Psychiatry, Department of Neurosciences/Department of Experimental and Clinical Neurosciences (DIMSC), Polytechnic University of Marche, Ancona, Italy

## Abstract

**Introduction:**

An increasing number of studies have investigated the role of inflammation in mood disorders, like an altered C-reactive protein (CRP) hematic level. Some studies have also shown an association between suicidal behavior and increased CRP levels.

**Objectives:**

The objective of this study was to evaluate the association between specific clinical features and high sensitivity CRP (hsCRP) levels with suicidal risk in mood disorders inpatients.

**Methods:**

A naturalistic, observational, cross-sectional study was carried out by retrospectively recruiting 353 adult inpatients affected by severe mental illness (SMI), excluding patients affected by inflammatory pathology, alcohol/substances use disorders or treaded by anti-inflammatory/immunosuppressive therapy. In this sample 241 patients suffering from mood disorders were selected. HsCRP levels were measured at the ward admission. All patients were assessed with subscale 5 of the Mini International Neuropsychiatric Interview (MINI-5-s), TEMPS-M, BPRS, HAM-D21, YMRS, CGI-S, CGI-I, MOCA, MDQ, MSRS.

**Results:**

A logistic regression analysis was performed to ascertain the effects of hsCRP and personality trait on the likelihood of suicidality risk. The logistic regression model was statistically significant, χ2(2) = 32.868, p < 0.001. The model explained 18.7% (Nagelkerke R2) of the variance in subjects with a suicidality risk and correctly classified 76.8% of cases. According to the logistic regression model, suicidality risk is negatively predicted by the total score of the YMRS (exp(B)=0.969, IC95%=0.947-0.993, p=0.01) and hostility subscale of the BPRS (exp(B)=0.905, IC95%=0.819-1.000, p=0.05) while it is positively predicted by the cyclothymic temperament subscale of the TEMPS-M (exp(B)=1.066, IC95%=1.017-1.118, p=0.008) and hsCRP (exp(B)=1.090, IC95%=1.012-1.174, p=0.024).

**Conclusions:**

The study suggests the potential transdiagnostic association between low grade inflammation, temperament and suicidal risk in patients affected by mood disorders. Our preliminary findings could support a routine introduction of hsCRP hematic measurement, due to its relatively low cost, for its possible utility as an early trans-diagnostically biomarker for suicidal risk. The findings could also lead to developing a model to identify subjects who may benefit from a combined anti-inflammatory and psychopharmacological treatment strategy during the acute illness phase. A neuroinflammatory approach could further help stage and subtype mood disorder patients in more homogenous samples and investigate short- and long-term treatment implications, clinical course, and prognosis. Further research studies should consider all illness phases and how specific temperament and chronotype may influence treatment response, illness course, and outcomes.

**Disclosure of Interest:**

None Declared

